# What are the consequences of over and undertreatment of type 2 diabetes mellitus in a frail population? A systematic review

**DOI:** 10.1002/edm2.470

**Published:** 2024-02-27

**Authors:** Helen O'Neil, Adam Todd, Mark Pearce, Andrew Husband

**Affiliations:** ^1^ School of Pharmacy Newcastle University Newcastle UK; ^2^ South Tyneside and Sunderland NHS Foundation Trust Sinderland UK; ^3^ NIHR North East and North Cumbria Applied Research Collaboration (NIHR NENC ARC) Newcastle UK; ^4^ Population Health Sciences Institute, Newcastle University Newcastle upon Tyne UK

**Keywords:** diabetes, frailty, glycaemic control

## Abstract

**Aims:**

This review aims to identify the evidence base for the consequences of over and undertreatment of type 2 diabetes mellitus in a frail population.

**Method:**

In this systematic review, we searched MEDLINE, Embase, PubMed, CINAHL and the Cochrane Library for studies from January 2001 to 15th August 2022. We included a variety of study types that assessed and reported frailty including patients ≥18 years old. Studies included those that reported the prevalence of over or undertreatment of diabetes mellitus in a frail population and those examining outcomes related to glucose control in frail older people living with diabetes. Data were extracted using a bespoke extraction table using a narrative synthesis approach.

**Results:**

A total of 4114 articles were identified with 112 meeting inclusion criteria. These included 15,130 participants across the 11 studies with sample sizes ranging from 101 to 11,140. Several areas were identified in the included studies where under or overtreatment of diabetes impacted outcomes for patients. These included hospital admissions, readmissions, length of stay, falls, mortality, cognitive impairment and cardiovascular disease outcomes.

**Conclusion:**

The results showed that there was a high heterogeneity of outcomes between the studies and that many examined small numbers of participants. In this review, both over and undertreatment were shown to increase adverse outcomes in frail older people. Further research around optimal glycaemic control for frail older people living with diabetes is required with the aim to identify ideal target ranges and produce practical clinical guidelines to promote attainment of these.

## Graphical abstract

This paper reviews the evidence base for the consequence of over and undertreatment of type 2 diabetes mellitus in a frail population with the aim to establish evidence‐based targets for blood glucose control in this population. The results showed that there was a high heterogeneity of outcomes between the studies and that many examined small numbers of participants. Both over and undertreatment were shown to increase adverse outcomes in frail older people, and further research is required to determine optimal glycaemic control for frail older people living with diabetes.

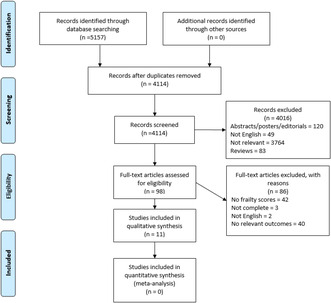

## 1 INTRODUCTION

Diabetes is one of the most common long‐term health conditions in the UK.^1^ In 2021–2022, it was estimated that over 4.3 million people in the UK have a diabetes diagnosis, with around 90% of cases being type 2 diabetes.^2^ Ageing and diabetes are risk factors for functional impairment.^3^, ^4^ Older people with diabetes have higher rates of premature death, functional disability, co‐existing illnesses and are at greater risk of developing geriatric syndromes, including delirium, falls and frailty.^5^ A recent meta‐analysis showed frailty was more prevalent in older adults with diabetes than those without.^6^

Frailty can be described as a complex interplay of health and illness, attitudes, resources and dependence on others, which leads to a decreased ability to withstand illness without loss of function.^7^ Frailty causes increased vulnerability with poor resolution of homeostasis after stressor events, which increases the risk of adverse outcomes occurring after seemingly minor events.^8^, ^9^ Failure to detect and consider frailty in older people results in exposure to interventions and treatments that could potentially cause more harm than potential benefit.^9^ The prevalence of both diabetes and frailty increases with age.^2^, ^10^, ^11^ It has also been demonstrated that diabetes is a risk factor for the development and progression of frailty.^12^

‘Treat to target’ is an important concept in diabetes management. Treat‐to‐target is a therapeutic concept that considers well‐defined, clinically relevant and specific end targets such as a blood pressure target or HbA1c target with the aim of controlling the pathophysiology of the disease.^13^ As people become more frail, these treatment targets are often not appropriate, and this can lead to overtreatment. Despite the recognition of this issue in frail patient living with diabetes, studies suggest that overtreatment is still common.^14^

Overtreatment of diabetes can lead to recurrent hypoglycaemia, which, in turn, causes cognitive impairment, falls and increased risk of mortality.^15^, ^16^ Alongside this, dementia and cognitive decline, which increases in incidence as people age, can negatively impact patients' ability to recognise hypoglycaemia and self‐manage diabetes.^16^ Hypoglycaemia is also often underdiagnosed in the older population due the non‐specific symptoms in this age group such as dizziness, feeling generally unwell and tiredness.^17^ By reducing overtreatment in diabetes, the risk of hypoglycaemia and the adverse events associated with it are minimised.

Conversely, whilst overtreatment is a problem, the consequences of undertreatment should not be underestimated and so hitting just the right level of glucose control between the two extremes is paramount. High blood glucose levels are associated with symptoms of polyuria, polydipsia and nocturia; in older adults, there is an association with increased risk of infection, hospitalisation, increased cardiovascular events and mortality.^14^ The key in diabetes management, therefore, is to balance treatment and ensure that patients are maintained at a blood glucose target that strays neither into under or over treatment.

There have been several national and international consensus and opinion guidelines published that provide frameworks for improving diabetes care in older populations.^5^, ^14^ These recommend glycaemic targets for patients based on the presence and severity of frailty.

## 2 METHOD

### 2.1 Aim

To review the evidence base for the consequence of over and undertreatment of type 2 diabetes mellitus in a frail population with the aim to establish evidence‐based targets for blood glucose control in this population.

### 2.2 Search Strategy and Eligibility Criteria

Methods were prespecified and reported according to Preferred Reporting Items for Systematic Reviews and Meta‐Analyses (PRISMA) guidelines. This study was registered with the international database of prospectively registered systematic reviews PROSPERO (identification number CRD42022346032).

**FIGURE 1:**
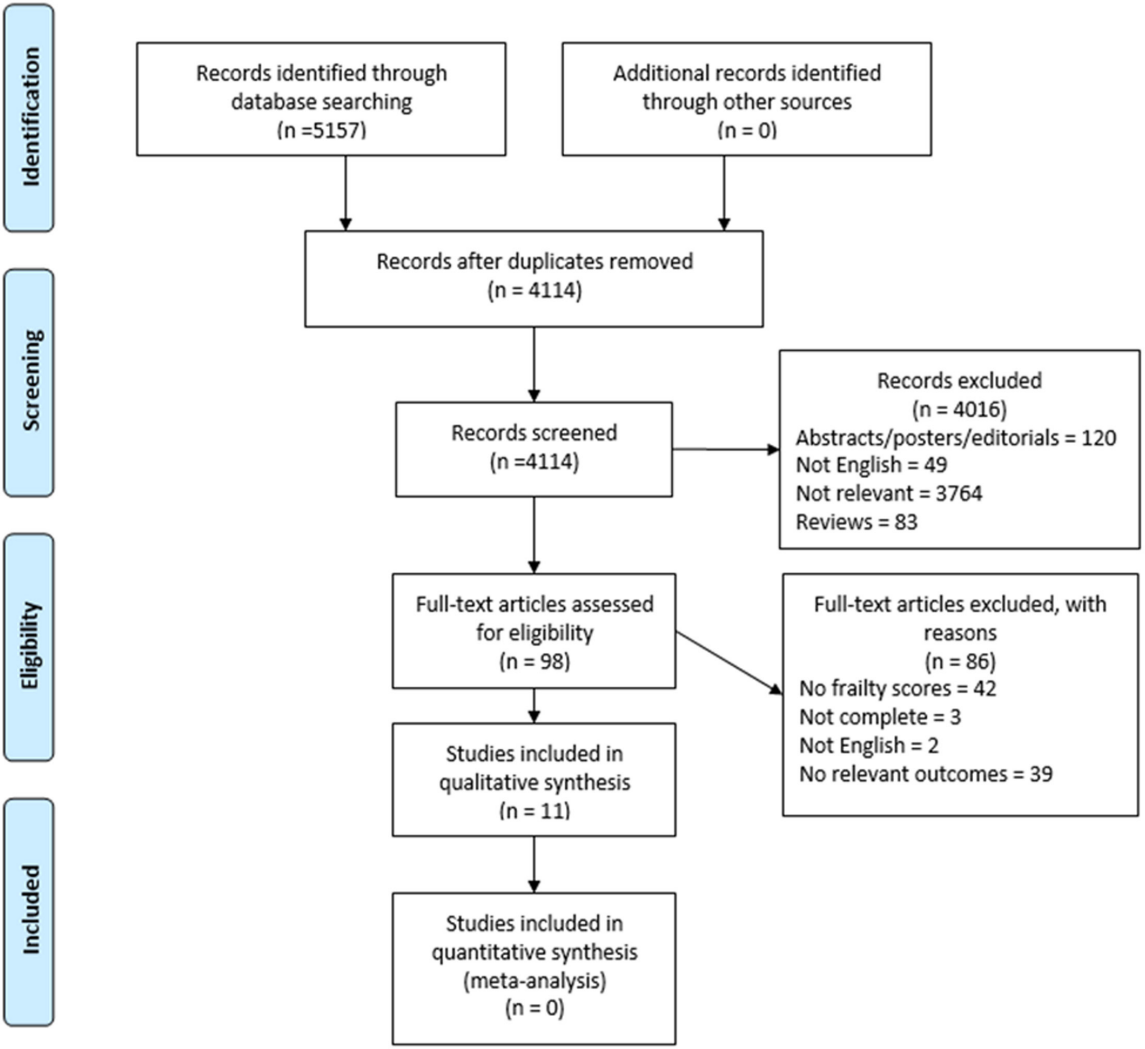
Prisma diagram of study selection.

Inclusion criteria were cross‐sectional observational studies, cohort studies, and longitudinal studies, in any care setting, in patients ≥65 years old. Studies included reported the prevalence of over or under treatment of diabetes mellitus in a frail population and studies examining outcomes related to glucose control in frail older people living with T2DM. Detailed inclusion criteria can be seen in the study protocol.^18^ The outcomes of interest in these studies were the clinical outcomes associated with either hypoglycaemic events and/orhyperglycaemia and the impact of these. No papers were excluded on the basis of quality (Figure 1).

Case series and case reports were excluded from the review, owing to the high potential for bias in these study designs. Only English language studies were included, and we have stated the number of papers excluded on basis of language at each stage.

The following databases were searched in August 2022, using a combination of Medical Subject Headings (MeSH) and keyword searches: MEDLINE, Embase, PubMed, CINAHL and the Cochrane Library. The search was structured using terms related to ‘frailty’, ‘type 2 diabetes’, ‘overtreatment’ and ‘undertreatment’ (see Appendix 1 for the search strategy). Endnote® was used to manage the search hits. After deduplication, titles and abstracts were screened, and then full texts articles were assessed for eligibility. We searched from 2000 to present, as the original papers standardising the definition of frailty were published in 2001. Database searches were supplemented by a manual search of reference lists of related papers and review articles and by forward and backward citation searching of all relevant studies.

All stages of screening, data extraction, and quality assessment were done by one author (HO), with checking by a second author (AH, AT or MP). Discrepancies were resolved by discussion and, if necessary, consensus with a third author.

## 3 DATA ANALYSIS

Data were extracted (HO) from the identified studies using a narrative synthesis approach. A bespoke data extraction table was developed for this process (see Table 1) and approved by all the authors. Once data extraction was complete, it was reviewed and verified by another author (AH/AT/MP).

**TABLE 1:** Recommended therapeutic targets and treatment de‐escalation thresholds (Adapted from Strain et al.).^14^

	De‐escalation threshold	Treatment target
The fit older adult with diabetes	53 mmol/mol (7.0%)	58 mmol/mol (7.5%)
Moderate – Severe frailty	58 mmol/mol (7.5%)	64 mmol/mol (8.0%)
Very severe frailty	64 mmol/mol (8.0%)	70 mmol/mol (8.5%)

## 4 CRITICAL APPRAISAL

Papers were quality assessed and critically appraised using Joanna Briggs Institute critical appraisal checklists for assessing the risk of bias of cohort or cross‐sectional studies. The tools include an 8‐ or 11‐item checklist and an overall rating that classified the quality of the studies.

## 5 ETHICS

Ethical approval was not required for this work.

## 6 RESULTS AND DISCUSSION

After deduplication, there were 4114 records, which were then screened by title and abstract. We assessed 98 full‐text articles for inclusion and identified 11 studies that met our inclusion criteria.^19^, ^20^, ^21^, ^22^, ^23^, ^24^, ^25^, ^26^, ^27^, ^28^, ^29^ These included 15,130 participants across the 11 studies with sample sizes ranging from 101 to 11,140 participants. Eight of the studies were observational cohort studies^19^, ^20^, ^21^, ^23^, ^25^, ^27^, ^28^, ^29^ and two were cross‐sectional studies^19^, ^20^ with a mix of prospective^19^, ^20^, ^21^, ^22^, ^23^, ^24^, ^25^ and retrospective^19^, ^21^, ^25^ studies. One study was post‐hoc analysis of a large randomised controlled trial.^26^

A range of care settings were included with 6 studies^20^, ^21^, ^24^, ^25^, ^26^, ^29^ from primary care settings, 4 from hospital inpatients^22^, ^23^, ^24^, ^27^ and 1 from a hospital outpatient department.^19^ Studies were from a wide range of geographical locations 3 from the USA,^25^, ^27^, ^28^ with 2 each from Italy,^20^, ^24^ Spain^21^, ^22^ and 1 each from the UK^23^ Australia,^26^ France^19^ and the Netherlands.^29^

Four studies specifically related to type 2 diabetes,^21^, ^23^, ^26^, ^27^ with the others only specifying a general diabetes diagnosis rather than type of diabetes. In most of the studies, all the participants were older people with diabetes (>64 years). Several studies, however, the participants were aged greater than 18 years but included older patients (>54 years) as a sub‐group.

A wide variety of frailty measures (either validated or well described) were used to classify participants as frail in the included studies. Most commonly, the Fried Frailty Phenotype Model^30^ or the Clinical Frailty Scale, a cumulative deficits model^31^ were used, with other scores closely relating to either of these models. An earlier paper^28^ from 2007 did not use either of these models but rather VES‐13^32^ likely due to the fact frailty scoring was much less prevalent in healthcare at that time.

The data in the studies was collected from 2001 through 2021. As original papers standardising the definition of frailty were not published until 2001, we limited our search to 2000 onwards.

In nine of the studies, the main aims were to investigate how glycaemic control impacted on adverse outcomes in an older population with diabetes, albeit examining a variety of different outcomes ranging from all‐cause mortality to cognitive impairment.^19^, ^20^, ^21^, ^23^, ^24^, ^25^, ^26^, ^28^, ^29^ For the other 2 studies, this was not the primary focus, but the data collected aligned with the inclusion criteria of this review.

Given the small number of studies found, it was decided to include studies that did not define the type of diabetes the participants in the study had. Given the usual prevalence data of older people with diabetes, it is likely that the majority of patients included in these studies would be likely to have type 2 diabetes.

The high heterogeneity that characterised the outcomes of the included studies did not allow us to perform a meta‐analysis of the results.

There were a number of areas identified in the included studies where under or overtreatment of diabetes impacted outcomes for patients. These included hospital admissions, readmissions, length of stay, falls, mortality, cognitive impairment and cardiovascular disease outcomes, which are discussed in detail below.

### 6.1 Hospital admission/service utilization/readmission

Four studies evaluated the impact of glycaemic control on either hospital admission or health care service utilization.^19^, ^20^, ^21^, ^27^ In one study^21^ there was no detailed data only a descriptive record in the results of a participant having an emergency room visit related to hypoglycaemia. In the other studies,^19^, ^20^, ^27^ frailty was associated with increased hospital admissions, readmission and increased service utilization. This is something that has been shown in previous studies including participants with numerous different co‐morbidities^33^, ^34^ and highlights the importance of frailty identification and management if we are able to address these issues. Gual et al. and O'Neil et al.^23^ found participants with higher glucose levels were more likely to have increased readmission rates. Previous studies have also shown higher blood glucose to be associated with increased readmission rates.^35^, ^36^ These studies showed the combination of frailty and hyperglycaemia increased the risk of readmission and service utilisation, evidencing that, although strict glycaemic control is to be avoided to reduce the risk of harm, hyperglycaemia is also to be avoided, emphasising the need for an appropriate target between the two extremes.

### 6.2 Length of hospital stay

Two studies^23^, ^24^ investigated length of hospital stay as an outcome although this was a secondary outcome in both studies. Paterni et al.^24^ showed that patients with diabetes faced significantly longer hospital stays than patients without diabetes but did not offer data for relationship to glycaemic control. In the study by O'Neil et al.^23^ poor glycaemic control was associated with a significant increase in length of stay. Like readmission rates, length of stay has previously been shown to be increased with higher admission blood glucose, reflecting the finding of the two studies included in this review.^35^

### 6.3 Mortality

Four of the studies examined mortality as an outcome.^19^, ^20^, ^26^, ^28^ Frailty was shown to be associated with an increased risk of all‐cause mortality in participants with diabetes in four of the studies.^21^, ^24^, ^26^, ^27^ Reflecting the Cacciatore et al.^37^ study, which concluded that clinical frailty significantly predicts mortality in subjects even without diabetes, although mortality rates were still higher in those with both frailty and diabetes. Ferri‐Guerra et al.^27^ found that frailty was significantly associated with higher all‐cause mortality in participants with previous hospitalisations. A study by Hodgson et al.^38^ found mortality was greatest in those with most frequent contacts with primary care. These two studies may reflect that those with increased service utilisation may well have the greatest health needs, but they can also suggest where further focus on care is required. Paterni et al.^24^ found that frail participants without optimal glycaemic control determined by HbA1c showed a four‐ to five‐fold higher mortality compared to robust participants, whilst no difference in mortality rate was found between robust and frail participants with strict glycaemic control (HbA1c < 48 mmol/mol). Earlier research reflected the same increase in mortality showing higher admission blood glucose to be associated with increased mortality.^35^ A previous study analysing the relationship of glycaemic control and mortality in older participants described a U‐shaped curve with low and high mean HbA1c values associated with increased all‐cause mortality and cardiac events.^39^ The population in this study was not categorized as frail or non‐frail; further evidence is needed to ascertain the optimum glycaemic target for frail people living with diabetes to improve mortality rates.

### 6.4 Hypoglycaemia

Four studies^19^, ^22^, ^26^, ^29^ assessed hypoglycaemia as an outcome. Paterni et al.^24^ recorded that 61.6% of participants taking hypoglycaemia‐inducing drugs suffered at least one symptomatic hypoglycaemic episode during their inpatient stay, but did not relate this to outcomes of the study. Mange et al.^19^ found no significant difference between suspected or confirmed hypoglycaemia rates between frail and non‐frail participants, whereas Nguyen et al.^26^ found severe hypoglycaemia was more common in frail participants. The study by Hart et al.^29^ showed that hypoglycaemia occurred in 20.3% of frail participants who were considered to be overtreated according to their HbA1c; however, the study numbers were very small. Molist‐Burnet et al.^22^ found hypoglycaemia was only noted occasionally and had only been recorded for 3 patients (1.42%), given reported hypoglycaemia rates in the literature this seems to be an under estimation. Mange et al.^19^ noted 9.1% of patients in their study had reported hypoglycaemia; however, as this was self‐reported, they felt it was highly likely the incidence was underestimated. Hypoglycaemia in a frail older population can cause serious harm and the disparity in the study results may be due to the poor recognition of hypoglycaemia in this population.^16^ As previously stated, hypoglycaemia is often underdiagnosed in an older population and is known to cause significant harm.^15^, ^16^, ^17^ Hart et al.^29^ noted that despite patients experiencing regular hypoglycaemia and related harm, treatment was not de‐intensified. This risk of hypoglycaemia has led to consensus guidelines whereby the HbA1c lower limit is raised in frail patients to reduce the risk of hypoglycaemia.^40^ Recognition of the need for individual targets to avoid hypoglycaemia in frail older people is becoming more common, but there is still further work to do to ensure this is being achieved in clinical practice. Use of new technologies such as using continuous glucose monitoring (CGM) to assess for hypoglycaemia is another emerging area that would be useful to expand research in. The use of CGM would remove the unpredictability of self‐reporting and can also be particularly useful in patients with cognitive impairment who are unlikely to recognise hypoglycaemia.^41^

### 6.5 Falls

Five studies^19^, ^23^, ^24^, ^26^, ^29^ reviewed the impact of glycaemic control in relation to falls. The primary focus of Nelson et al.^28^ was glycaemic control and falls, and they concluded HbA1c levels below 7% (53 mmol/mol) were significantly correlated with falls, but only when the cohort was reviewed as a whole sample rather than just the frail group. The authors felt this was due to small sample size in the subgroup analysis and an already greater risk of falls in the frail population. The study showed it was likely that low HbA1c was a risk factor for falls in an elderly population regardless of frailty status. O'Neil et al.^23^ found that patients with low HbA1c were more likely to be admitted to hospital with a fall with 69% (*n* = 22) of participants admitted with a fall having a low HbA1c. Hart et al.^29^ found that 25% (*n* = 16) of patients who were overtreated reported accidents involving falls. Mange et al.^19^ found in their study that 47.8% of patients who had a fall recorded also had a low HbA1c. Falls were also seen in those who did not necessarily have a HbA1c below target but were taking hypoglycaemic agents (insulin, sulphonylureas) with 41% (*n* = 9) of patients who had fallen in the O'Neil study^23^ and 33% (*n* = 12) in the Mange study.^19^ Paterni et al.^24^ reported that participants with lower levels of HbA1c also more frequently experienced syncopal falls and felt this could be explained by the association between falls and hypoglycaemia. Falls are often multifactorial in a frail population, but hypoglycaemia has been shown to increase fall risk in older people.^42^ Alongside, this Type 2 diabetes has also been shown to increase fall risk as well as increasing bone fragility, therefore increasing fracture risk.^43^ Given the increased falls risk in a frail population as well as the significant harm that can result from a fall, we must ensure we look at areas where we can actively reduce that risk such as prevention of hypoglycaemia.

### 6.6 Cognitive impairment

Mone et al.^20^ examined how glycaemic control impacted cognitive impairment and found a strong correlation between the Montreal Cognitive Assessment (MoCA) scores and blood glucose levels (*p* < .001) showing participants with hyperglycaemia had a lower MoCA score, identifying worse cognition. Zaslavsky et al.^25^ investigated the association of HbA1c levels with cognitive function and found that moderate levels of HbA1c (53‐64 mmol/mol or 7%–8%) were associated with better cognition in people in their early 80s, but this did not translate to people in their late 80s and early 90s. Frailty did not affect the association between HbA1c and cognitive score. The study also looked at gait speed, but in frail participants, no difference in gait speed was found between the HbA1c values. The studies included were small and point to the lack of evidence we currently have in this area. Other studies have found that both hyperglycaemia and hypoglycaemia contribute to worsening cognitive impairment further adding weight to the argument that we should have a target range rather than just aiming to avoid hypoglycaemia in this population.^44^, ^45^, ^46^ It has also been shown that cognitive impairment is detrimental to a person's ability to not only self‐manage their diabetes but also impairs their ability to recognise hypoglycaemia, which can then in turn further worsen their cognitive impairment.^16^

### 6.7 Macro micro‐vascular outcomes

Nguyen et al.^26^ were the only study in this review to look at micro‐ and macro‐vascular complications in study outcomes. The post‐hoc analysis by Nguyen et al.^26^ found that frail participants had a higher incidence of micro‐ and macro‐vascular events (combined or alone) and showed that intensive glucose control was less effective in frail participants compared to non‐frail for prevention of combined micro and macro‐vascular events. This evidence suggests that less stringent blood glucose targets would be appropriate in frail older population living with diabetes. However, we need further reliable evidence to assess which glucose levels are the safest and most effective in prevention of micro and macro‐vascular outcomes whilst avoiding harm caused by hypoglycaemia.

## 7 STRENGTH AND LIMITATIONS

This review is the first to focus on reviewing the impact of glycaemic control on outcomes in frail older people living with diabetes. A thorough search of several large databases was completed using standardised methods for both searching and reviewing eligible studies. Despite this, there were several limitations to this review. Only those studies in English were included. Most studies included contained small numbers of participants, and the high heterogeneity of the study samples and the varied outcomes made it difficult to compare the studies. The relationship of glycaemic control to the outcome of the studies was also not always the primary focus, meaning that it was not easy to see the relationship between glycaemic control and certain outcomes, despite them being focused on in the study.

## 8 BIAS

The risk of bias for the studies is reviewed and highlighted in the data extraction table (Table 2).

**TABLE 2:** Data extraction table.

First author	Year	Country	Design	No.Pts	Age (mean)	Female (%)	Frailty Score method	HbA1c	Care setting	Main objectives of study	Outcomes of interests	Main results	Bias
Ferri‐Guerra^27^	2020	USA	Retrospective cohort	763	72.9	1.7	Frailty index Deficit accumulati‐on	<7421 7–9265 >9 77	Community Outpatients	To determine the association of frailty with all cause hospitalisations and mortality in older veterans with diabetes mellitus (DM)	Hospitalization Mortality	Frailty significantly associated with increased risk of all cause hospitalization (*p* < .0001) and mortality (*p* = .014)	Doesn't relate to treatment ranges despite being shown in characteristics. Frailty only assessed electronically. Only frail or non‐frail
Gual^21^	2019	Spain	Prospective observational cohort	532	84.3	38.9	FRAIL scale	100–124 mg/dL >125 mg/dL	Hospital	Assessment of the prognostic impact of diabetes according to frailty status in acute coronary syndromes	Mortality Readmission at 6 months	Participants with DM had significantly higher incidence of mortality or readmission within 6 months, significant in participants with frailty (*p* = .030). Patients with glucose levels >125 mg/dL had significantly higher incidents of mortality or readmission compared to normo‐glycaemic participants (*p* = .049)	Unclear link between glycaemic control and outcomes frailty specific
Hart^29^	2018	Netherla‐nds	Observational cohort	319	76.6	48.3	Frailty index	53.3	Primary care records	To assess level of personalized care in pts with type 2 DM focusing on overtreatment	Hypoglycaemia ED attendance Falls Polypharmacy	40% of older adults are overtreated	Very unclear study Small numbers
Mange^19^	2021	France	Cross‐sectional observational cohort	110	81.7	61.8	Fried criteria	7.11%	Outpatients	Evaluation of older people with diabetes whose treatment deviates from recommendations	Falls Polypharmacy Hypoglycaemia	52% of frail patients are overtreated, significantly higher than non‐frail counterparts. Patient who have fallen almost 1/3 had glycaemic control that was too strict. 60.9% had more than one drug therapy problem	Single centre Cross‐sectional Small numbers Only frail or non‐frail Patient reported hypoglycaemia
Molist‐Brunet^22^	2019	Spain	Prospective observational	210	86.1	55.2	Frail‐VIG index	7.28%	Hospital	Prevalence of Type 2 DM in frail patients Identification of inappropriate prescriptions for antidiabetic drugs Evaluate link between polypharmacy and frailty degree	Polypharmacy Hypoglycaemia Inappropriate prescriptions (IP)	93.3% of Patients had polypharmacy IP identified in 66.2% of patients, 69.8% of IP was overtreatment (46.2% of pts). Increasing frailty increasing polypharmacy (*p* < .05) IP increases with increasing frailty, in‐particular significant in overtreatment (*p* < .01)	Small numbers *p* values not quoted exactly Hypoglycaemia only from clinical notes – small number reported
Mone^20^	2021	Italy	Prospective observational	209	75 (Normoglycaemic) 76 (hyperglycaemic)	Not recorded	Fried criteria		Primary Care	Effects of hyperglycaemia and metformin on cognitive impairment in frail hypertensive patients	Cognitive impairment (MoCA score)	Hyperglycaemic patients have a lower MoCA score than NG ones.(*p* < .05) Strong correlation between MoCA score and blood glucose levels (*p* < .001) MoCA score in metformin treated HG participants significantly different from insulin treated HG pts (*p* < .0001)	Focus hypertension Didn't look at overtreatment Small numbers
Neslon^28^	2007	USA	Retrospective case‐controlled	111	78.1 (Frail) 78.5 (non‐frail)	63.2 (frail) 35.2 (non‐frail)	VES‐13	7.44 mean 45.6% below 7% (frail) 7.61 mean 37% below 7% (non‐frail)	Primary care – recruited to study via letter	To determine if glycaemic control contributes to falls risk in frail and non‐frail adults with DM	Falls	HbA1c <7.0 correlated to increasing falls (*p* = .001) neuropathy was also correlated (*p* = .006)	Telephone frailty scoring Older paper with less recognized frailty screening Small numbers in frail group Exclusion of most frail and vulnerable, dementia pts excluded
Nguyen^26^	2021	Australia	Post hoc analysis of ADVANCE RTC	11,140	65.8 Measure affects in those below 65 years and those above in separate analysis	25.7	Frailty index based on Rockwood accumulative deficit model	7.52%	RTC – community	To develop a frailty index and explore the relationship of frailty to adverse outcomes on the effectiveness of more intensive blood glucose and BP control in those with type 2 DM	Micro and macro‐vascular events All‐cause mortality Cardiovascular mortality Severe hypoglycaemia Hypotension/dizziness	Frail participants had higher incidents of macro and micro‐vascular events, all‐cause mortality, CVD mortality, and severe hypoglycaemia Intensive glucose control was more effective in non‐frail participants compared with frail. Severe hypoglycaemia more common in frail participants (*p* = .001)	Retrospective frailty scores with own index Post‐hoc analysis
O'Neil^23^	2022	UK	Prospective observational cohort	101	Pre/mild frailty 82.2 Mod frailty 83.1 Severe frailty 81.3	Pre 43% Mod 60.5% Severe 64%	Frailty Index Rockwood	Pre 54.8 Mod 56.1 Sever 67.8	Hospital	To assess if frail patient with diabetes are being treated to the correct blood glucose targets and observe harm of over or undertreatment	Overtreatment Undertreatment Polypharmacy Length of stay Readmission rates	61% of patient deemed to be overtreated with 46% taking anti‐hyperglycaemic agents 28% of patients were undertreated 96% of patients had polypharmacy with 71% taking over 10 medications 69% of patients admitted with a fall were overtreated Those with higher HbA1c had significantly longer length of stay (*p* = .036) Undertreated patients were more likely to be readmitted within 30 days (*p* = .008)	Small numbers Single centre
Paterni^24^	2021	Italy	Prospective observational cohort	1319	82.8	75.9	Clinical Frailty Scale Rockwood	3 groups <48 48–58 >58	Hospital	To investigate prognostic role of HbA1c and frailty level in older DM patients admitted with hip fracture	Mortality Length of stay Polypharmacy	Patients with low HbA1c showed higher prevalence of syncopal falls (*p* + .05) Frail type DM patients showed significantly higher mortality compared to robust ones (*p* = .001) Frailty is an independent mortality predictor for individuals with a HbA1c above 48 mmol/mol	Single centre Low number of frail patients Focus on hip fracture patients Only frail or non‐frail
Zaslaysky^25^	2020	USA	Prospective cohort	316	83	55–66% dependent of HbA1c	Adapted fried criteria	<7% 7%–8% >8%	Primary Care	Association between glycaemic control (HbA1c) level and cognitive and physical function	Cognitive impairment Gait speed	HbA1c levels of 7–8% had higher cognitive scores. This difference diminished when people were in their late 80's/early 90's Elevated HbA1c >8% were associated with worse functioning in terms of slower gait speed.	Small numbers Missing data

## 9 CONCLUSION

The results in this systematic review showed that there was a high heterogeneity of outcomes between the studies and that many examined small numbers of participants. This is not unexpected given that older people were often previously excluded from clinical trials and diagnosing frailty using a validated tool is only something that has occurred in recent years. However, in this review, both over and undertreatment were shown to increase adverse outcomes in frail older people. In recent years, it has become more widely accepted that hypoglycaemia in a frail population should be avoided to reduce harms, although, as shown in some of the studies, people with a low HbA1c were not de‐intensified, showing clinic inertia. Hyperglycaemia in frail older people can also cause significant harm, although there seems to be less focus on a reduction of this. Further research around optimal glycaemic control for frail older people living with diabetes is required with the aim to identify ideal target ranges and produce practical clinical guidelines to promote attainment of these.

Across all studies, frailty was linked to inferior outcomes, and given that studies have shown people living with diabetes are more likely to become frail, this is something we should be actively assessing for in older people living with diabetes. Frailty does not show a linear trajectory and can be slowed or reversed with the correct treatment, and therefore, early identification of frailty risk is paramount. The management of diabetes itself can also impact frailty risk and the likelihood of adverse outcomes. Frailty gives the conceptual basis to provide a more holistic, person‐centred focus for care of people living with both diabetes and other multi‐morbid conditions. Currently, the only practical guidelines of how to manage diabetes in frail older people are consensus guidelines This review reflects this lack of evidence, highlighting the need for further research in this population to aid evidence‐based care.

## AUTHOR CONTRIBUTIONS

**Helen O'Neil:** Conceptulization (lead); validation (equal); formal analysis (lead); investigation (lead); writing – original draft (lead), writing – review and editing (equal); visualization (lead); project administration (lead); funding acquisition (lead). **Adam Todd:** Supervision (equal); validation (equal); writing – review and editing (equal). **Mark Pearce:** Supervision (equal); validation (equal); writing – review and editing (equal). **Andrew Husband:** Supervision (equal); validation (equal); writing – review and editing (equal).

## CONFLICT OF INTEREST STATEMENT

The authors have declared no conflicting interests.

## Data Availability

Data sharing not applicable to this article as no datasets were generated or analysed during the current study.

## APPENDIX 1 Search Strategy

### MEDLINE

1. frailty.mp. or Frail Elderly/ or Frailty/ 28,099.
2. frail.mp. 22,775.
3. older.mp. 501,766.
4. vulnerable.mp. 111,340.
5. 3 and 48,647.
6. 1 or 2 or 541,972.
7. type 2 diabetes.mp. or Diabetes Mellitus, Type 2/ 209,938.
8. diabetes.mp. or Diabetes Mellitus/ 700,771.
9. 7 or 8,700,771.
10. undertreatment.mp. 2951.
11. overtreatment.mp. or Overtreatment/ 5295.
12. Hyperglycemia/ or hyperglycaemia.mp. 38,130.
13. Hypoglycemia/ or hypoglycaemia.mp. 35,655.
14. HbA1c.mp. or Glycated Haemoglobin A/ 62,956.
15. glycated haemoglobin.mp. 4262.
16. blood glucose targets.mp. 87.
17. 10 or 11 or 12 or 13 or 14 or 15 or 16,132,846.
18. Mortality/ or mortality.mp. 1,307,294.
19. hospital admission.mp. 29,773.
20. Patient Readmission/ or readmission.mp. 36,868.
21. major adverse cardiovascular events.mp. 5591.
22. MACE.mp. 9819.
23. Falls.mp. or Accidental Falls/ 64,117.
24. Delirium/ or Delirium.mp. 21,452.
25. Hospitalization/ or hospitalization.mp. 142,335.
26. length of stay.mp. or “Length of Stay”/ 137,611.
27. cognitive impairment.mp. or Cognitive Dysfunction/ 82,907.
28. lower limb amputation.mp. 1726.
29. infection.mp. or Infections/ 1,366,866.
30. 18 or 19 or 20 or 21 or 22 or 23 or 24 or 25 or 26 or 27 or 28 or 292,884,741.
31. 17 or 303,002,358.
32. 6 and 92,007.
33. 31 and 321,082.

Duplicates removed 1080.
